# Understanding patient barriers and facilitators to uptake of lung screening using low dose computed tomography: a mixed methods scoping review of the current literature

**DOI:** 10.1186/s12931-022-02255-8

**Published:** 2022-12-23

**Authors:** Debbie Cavers, Mia Nelson, Jasmin Rostron, Kathryn A. Robb, Lynsey R. Brown, Christine Campbell, Ahsan R. Akram, Graeme Dickie, Melanie Mackean, Edwin J. R. van Beek, Frank Sullivan, Robert J. Steele, Aileen R. Neilson, David Weller

**Affiliations:** 1 Usher Institute, University of Edinburgh, Doorway 1, Medical School, University of Edinburgh, Teviot Place, Edinburgh, EH8 9AG UK; 2 The National Institute of Economic and Social Research, 2 Dean Trench Street, London, NW1P 3HE UK; 3 Institute of Health and Wellbeing, University of Glasgow, 1 Lilybank Gardens, Glasgow, G12 8RZ UK; 4 School of Medicine, University of St. Andrews, North Haugh, St. Andrews, KY16 9TF UK; 5 MRC Institute of Genetics and Molecular Medicine, University of Edinburgh, Edinburgh, UK; 6 Edinburgh Cancer Centre, Western General Hospital, Crewe Road South, Edinburgh, EH4 2XU UK; 7 Edinburgh Imaging, Queen’s Medical Research Institute, University of Edinburgh, 49 Little France Crescent, Edinburgh, EH16 4TJ UK; 8 School of Medicine, University of Dundee, Ninewells Hospital, Dundee, DD1 9SY UK

**Keywords:** Scoping review, Lung cancer screening, Mixed methods, Early detection, Respiratory health, Cancer screening

## Abstract

**Background:**

Targeted lung cancer screening is effective in reducing mortality by upwards of twenty percent. However, screening is not universally available and uptake is variable and socially patterned. Understanding screening behaviour is integral to designing a service that serves its population and promotes equitable uptake. We sought to review the literature to identify barriers and facilitators to screening to inform the development of a pilot lung screening study in Scotland.

**Methods:**

We used Arksey and O’Malley’s scoping review methodology and PRISMA-ScR framework to identify relevant literature to meet the study aims. Qualitative, quantitative and mixed methods primary studies published between January 2000 and May 2021 were identified and reviewed by two reviewers for inclusion, using a list of search terms developed by the study team and adapted for chosen databases.

**Results:**

Twenty-one articles met the final inclusion criteria. Articles were published between 2003 and 2021 and came from high income countries. Following data extraction and synthesis, findings were organised into four categories: *Awareness of lung screening*, *Enthusiasm for lung screening*, *Barriers to lung screening*, and *Facilitators or ways of promoting uptake of lung screening*. Awareness of lung screening was low while enthusiasm was high. Barriers to screening included fear of a cancer diagnosis, low perceived risk of lung cancer as well as practical barriers of cost, travel and time off work. Being health conscious, provider endorsement and seeking reassurance were all identified as facilitators of screening participation.

**Conclusions:**

Understanding patient reported barriers and facilitators to lung screening can help inform the implementation of future lung screening pilots and national lung screening programmes.

**Supplementary Information:**

The online version contains supplementary material available at 10.1186/s12931-022-02255-8.

## Introduction

Cancer is a leading cause of death and reduced life expectancy worldwide, with lung cancer the primary cause of cancer death [1, 2]. In 2020, there were a reported 2.1 million new lung cancer cases and 1.8 million deaths [3]. Lung cancer incidence and mortality are closely linked to smoking patterns and subsequent tobacco control programmes [4–6], and while smoking cessation is related to the highest reduction in mortality, detection at an early stage also offers the prospect of improved survival rates [7].

Historically, lung cancer detection largely arises through symptomatic presentation [8]. However, symptoms are often generic in nature and not considered serious at the time of their onset, with prolonged intervals from both symptom onset to help seeking, and from first help seeking to diagnosis [8–10]. Furthermore, lung cancer is predominately asymptomatic in the early stages, taking several years to reach the stage when it is most frequently diagnosed. Thus, advanced local disease and metastatic spread at diagnosis is commonplace, rendering the cancer less likely to be curatively treatable [11].

With the success of breast, cervical and bowel cancer screening, there has been an ongoing interest in establishing an effective equivalent programme for lung cancer [7]. While programmes based on chest X-rays or sputum cytology screening have not shown benefit, early detection via low-dose computed tomography (CT) has been trialled widely through North America and Europe and is now endorsed by the United States Preventative Services Task Force (USPSTF) [12]. Early trials demonstrated that more cancers, and specifically increased stage I cancers, could be identified by routine low-dose CT screening [13–15]. Recent studies have also shown survival benefit—but only when screening is successfully targeted towards at risk populations to minimise ineffective screening and over-diagnosis [7, 16, 17].

Worldwide, lung cancer screening is not consistently offered at a national level, with current provision taking the form of local pilots and programmes in Europe, and screening in the USA is often reliant on physician support for implementation [7]. For screening to achieve improved outcomes, it requires sufficient uptake in target groups; within the trial setting, initial uptake of screening has been notably low, in the range of 50–60% [18–21], although this is difficult to calculate accurately as not all people offered screening are eligible for LDCT. Moreover, uptake is higher in people who used to smoke and in higher socio-economic status (SES) individuals, and lower in those most at risk (i.e. people who have smoked long term in lower SES groups) [18, 22–24]. Patterns of low and socially skewed uptake have also been reported in settings where screening is offered as part of routine healthcare services [25].

This scoping review aimed to identify literature exploring barriers and facilitators to participation in lung screening using low-dose CT. We were particularly interested in identifying barriers related to issues of deprivation and rurality that may affect screening participation. This review forms part of a larger preparatory study including stakeholder consultation, focus groups and document review to inform the development of a pilot low-dose CT lung screening intervention to be tested in the eligible Scottish population (‘LungScot’)[26]. Findings from the review and subsequent pilot will inform the roll out of targeted lung screening in Scotland.

## Methods

The review used a scoping methodology with the intention of providing high quality evidence in a short timeframe [27–29]. The aims of the review were to identify the barriers and facilitators to participation in lung screening, to identify issues related to deprivation and rurality, and to inform the development of a subsequent pilot lung screening intervention. This chosen methodology facilitates insight to a broad topic area to understand the landscape and map key concepts, rather than focusing on a research question looking at effectiveness—thus better reflecting the aims and purpose of our review [27–29]. We followed Arksey and O’Malley’s five-step process including identifying the research question, identifying the studies, study selection, charting the data, and collating, summarising and reporting the results [27]. Reporting of the review follows the PRISMA extension for scoping reviews PRISMA-ScR [30, 31].

### Protocol and registration

The protocol for this study was developed as part of the full feasibility study by the LungScot team. It was subject to internal and external peer review during the full study design and funding application and discussed with the wider study advisory group including clinical and government experts in screening. The protocol was not registered or published (See Additional file 1).

### Eligibility criteria and dimensions of interest

Research studies were eligible for inclusion if they were published, in English, within the specified timeframe (January 2000-May 2021), in a peer reviewed journal, and reported the results of empirical research in full. This time period was chosen to reflect the growing interest in lung screening and advent of lung screening trials worldwide. We primarily sought studies reporting on barriers and facilitators from high-income countries that examined behavioural aspects of lung screening behaviour. Details of the eligibility criteria are listed in Box 1. Articles focused on non-UK health system specific factors (such as opportunistic physician referrals in the US health system) were excluded unless they also contained data on patient related barriers.

Box 1: eligibility for inclusion in the review

- Articles published in English in the listed databases between January 2000-May 2021
- Articles covering the dimensions of interest i.e. reporting studies of barriers and facilitators to low-dose CT lung screening in the relevant populations
- Primary empirical studies with a qualitative, quantitative or mixed methods design
- Articles published in peer-reviewed journals
- Any participant group regardless of age, gender or ethnicity

**Information sources**

MEDLINE, EMBASE, PsycINFO, Cumulative Index of Nursing and Allied Health Literature (CINAHL), Web of Science, ASSIA, and Sociological Abstracts databases were searched from January 2000 to May 2021. We additionally searched the reference lists of literature reviews and discussion papers identified via the database searches to find additional relevant studies.

**Search**

Initial search terms were developed and tested for sensitivity and specificity. These included, but were not limited to: Lung cancer OR lung neoplasm) AND Screening AND Barriers OR Facilitat$ OR Health literacy OR Socioeconomic OR Deprivation OR Candidacy OR Access$ OR Participation OR Eligibil$ OR Disprarit$. Search terms were subsequently refined and adapted for different databases.

The MEDLINE search strategy was developed by MN in consultation with DC and the wider project team, and was translated for use in other databases using appropriate syntax. An example of the full electronic search strategy is given in Box 2.

Box 2: electronic search strategy example (MEDLINE version)

1. Lung cancer.mp. or *lung cancer/ or *lung tumor/
*mass screening/ or *cancer screening/ or screening.mp.
3. limit 2 to (english language and yr = "2000 -Current")
4. 1 and 3
*diagnostic procedure/ or diagnosis/ or risk assessment/ or risk factors.mp.
6. ("high risk" or assess* or model* or tool* or "primary care" or "general practice" or implement* or algorithm or strateg* or "over diagnosis" or valid*).mp. or cancer risk/ or primary health care/ [mp = title, abstract, heading word, drug trade name, original title, device manufacturer, drug manufacturer, device trade name, keyword, floating subheading word, candidate term word]
7. 5 and 6
8. 4 and 7
9. (Implementation or uptake or recruit* or participat*).mp.
10. 8 and 9
11. (Barriers or Facilitators).mp.
12. 4 and 11
13. 10 or 12

**Selection of sources of evidence**

Papers were included in the review if they matched the inclusion criteria and dimensions of interest, with a focus on perceptions of barriers and/or facilitators to lung screening.

**Fig. 1:**
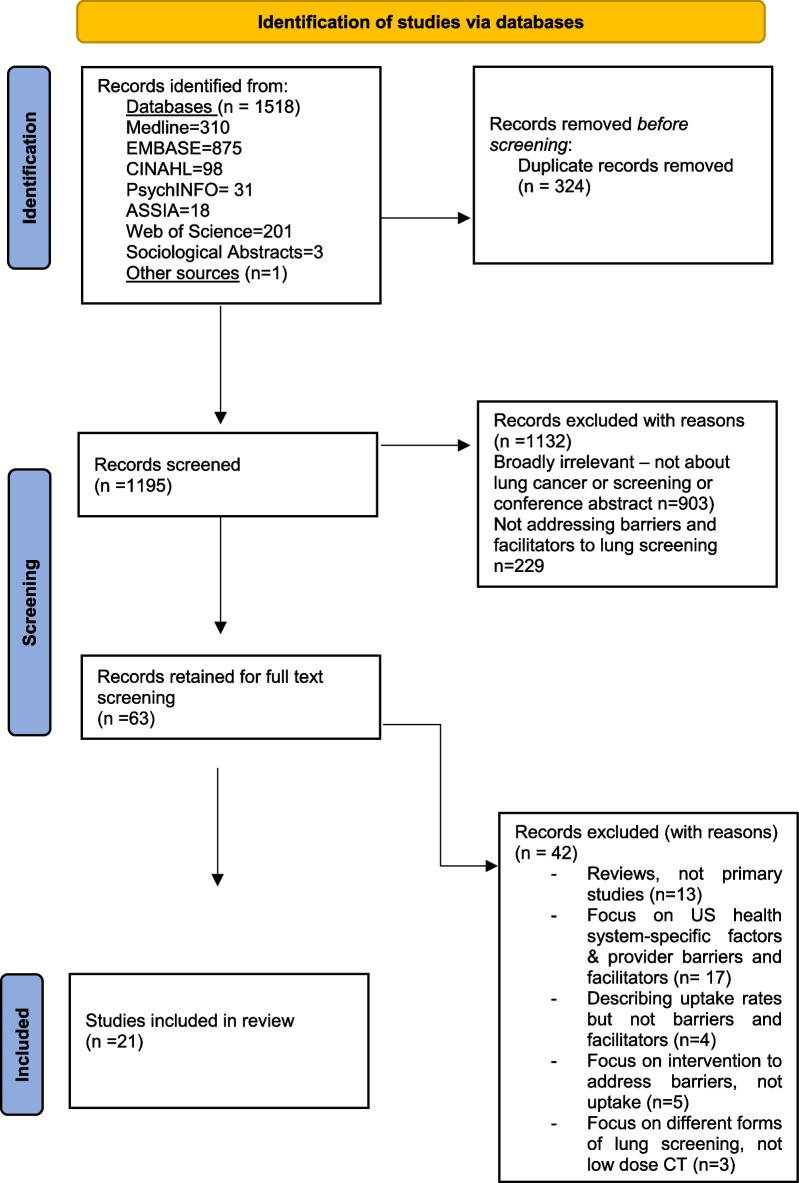
**PRISMA diagram showing screening process and study selection.** *Adapted From:* Page MJ, McKenzie JE, Bossuyt PM, Boutron I, Hoffmann TC, Mulrow CD, et al. The PRISMA 2020 statement: an updated guideline for reporting systematic reviews. BMJ 2021;372:n71. https://doi.org/10.1136/bmj.n71

Articles were subject to a three-stage screening process: (1) initial title screening to remove clearly irrelevant studies, (2) title and abstract screening to select potentially relevant studies, and (3) full text screening to select the final studies for inclusion in the review. The screening process was undertaken by two authors (DC and MN) and differences that could not be resolved were brought for discussion with the Principal Investigator (DW). An overview of the screening review and reasons for exclusion is given in Fig. 1. Final selection of articles took place after the data charting process described below, in discussion with authors DW, JR and LB. Consistent with a scoping review methodology, articles were not subject to critical appraisal of quality.

**Data charting process and data items**

Data were charted using a form developed by DC that was tested and refined by DC and JR during a pilot data extraction process. The information charted included:

- Author, year, journal, title
- Country of study
- Study type/design
- Methods, aims and objectives
- Participant characteristics, N
- Recruitment strategy
- Analysis
- Relevant findings
- Limitations
- Implications
- Notes

**Synthesis of results**

We undertook a thematic approach to synthesis in this review, drawing on guidance by Thomas and Harden [32, 33]. We considered using a purely narrative approach due to the heterogeneous nature of the included studies with mixed study designs. However, there was strong consistency in the findings across the studies that allowed us to identify common themes. Using an approach similar to that used in primary qualitative research—involving a descriptive summary of both qualitative and quantitative findings (thus effectively converting quantitative findings to a narrative or qualitative summary, similar to the technique used in critical interpretive synthesis [34])—we compared extracted findings across studies to form data codes. DC coded the extracted data using QSR Nvivo version 12 Pro (www.qsrinternational.com) and discussed and refined findings with DW and LB. From this, we were able to identify descriptive themes in findings across included studies. This approach has been used elsewhere [35, 36]. The themes relating to each included study are listed in Table 2.

**Patient and public involvement**

The design of this scoping review and protocol were shared with our LungScot patient advisory group for comment. The opportunity to comment on the extracted data and synthesis was also offered to the group, but no changes were made to the analysis as a result of this.

## Results

### Summary of included studies

We identified 21 papers for final inclusion in the review after consultation with the wider research team [37–57]. We excluded papers where findings did not address patient barriers and facilitators to low-dose CT lung screening. All papers originated from high income countries including the United States (US) (n = 15), United Kingdom (UK) (n = 5) and Australia (n = 1). There was a mix of qualitative (n = 8), quantitative (n = 10) and mixed methods studies (n = 3) included and articles were published between 2003 and 2020. Seventeen out of the 21 papers were published from 2017 to 2020, reflecting the fast-paced movement of lung cancer screening research in high income countries in recent years. Despite our intended focus on deprivation and rurality, few papers that met the inclusion criteria were found with content on these issues. Articles were a mix of prospective and retrospective studies, and study subjects included people who currently smoke, people who don’t smoke and people who used to smoke. Figure 1 shows the screening and selection process to arrive at this final set of studies.

Table 1 shows the summary characteristics of the included studies.

**Table 1:** Characteristics of included studies

Author, year, journal	Country of study	Study type/ method used	Participant characteristics	Reported analysis
1. Ali et al. 2015, BMJ Open (37)	UK	Mixed methods, cohort analysis and questionnaire	N = 6817 (748 completed the questionnaire), High risk individuals invited to the UKLS trial	Multivariate analysis plus thematic analysis of free text data
2. Carter-Harris et al. 2017a, Family Practice [38]	USA	Qualitative, semi-structured interviews	N = 18, people at high risk of lung cancer who have declined screening invitation	Thematic content analysis
3. Carter-Harris et al. 2017b, Health Expectations [39]	USA	Qualitative, focus groups	N = 26, long-term smokers eligible for lung screening, mix of those who have and haven’t been screened	Content analysis
4. Delmerico et al. 2014, Lung Cancer (40)	USA	Quantitative, telephone survey	N = 1290, representative sample of US adults aged 18 ±current, never and former smokers	Logistic regression
5. Draucker et al. 2019, Health Expectations [41]	USA	Qualitative, telephone semi-structured interviews	N = 40, people eligible for screening, mix of participants and non-participants in screening	Content analysis
6. Greene et al. 2019, Journal of Cancer Education [42]	USA	Qualitative, telephone semi-structured interviews	N = 37, recent participants in lung screening 55–74 years with a 30 year pack history	Iterative inductive content analysis
7. Jonnalagada et al. 2012, Lung Cancer [43]	USA	Quantitative, cross-sectional survey	N = 108, people eligible for lung screening aged 55–74 with a 10 pack year smoking history	Logistic regression
8. Lowenstein 2019, Lung Cancer [44]	USA	Qualitative, interviews	N = 42, screening eligible patients and a convenience sample of doctors from a primary care practice	Thematic content analysis
9. Percac-Lima et al. 2019, Journal of Immigrant and Minority Health [45]	USA	Quantitative, telephone survey	N = 460, 50–79 year old current and former smokers receiving follow up care at a community health centre	Logistic regression and principal components analysis
10. Quaife et al. 2017, Health Expectations [46]	UK	Mixed methods, survey and interviews	N = 184, people aged 40 + , smokers and former smoker with low socio-economic status	Chi squared and Fisher's exact test for survey findings and inductive thematic analysis
11. Quaife et al. 2018, BMC Cancer [47]	UK	Quantitative, national survey	N = 1445, general population of English adults age 50–70 who would be eligible or almost eligible for LCS	Chi squared and logistic regression
12. Raju et al. 2020, Clinical Lung Cancer [48]	USA	Quantitative, cohort analysis and survey with sub-set of patients	N = 818, participants in a retrospective analysis of those invited to lung screening at one hospital. Survey with sub-set of non-participants	Descriptive analysis, multivariate logistic regression, stepwise variable selection
13. Raz et al. 2019, Clinical Lung Cancer [49]	USA	Quantitative, survey	N = 185, current smokers attending a smoking cessation class	Descriptive statistics, chi-squared, univariate and multivariate logistic regressions
14. Roth et al. 2018, PLoS One [50]	USA	Qualitative, in-depth interviews	N = 20, men and women who had completed lung screening	Inductive content analysis
15. Schiffelbein et al. 2020, Journal of Primary Care and Community Health [51]	USA	Mixed methods, concurrent embedded design—survey and focus groups	N = 23, rural residing residents who met the US lung cancer screening eligibility criteria	Deductive and inductive analysis
16. Schnoll et al. 2003, Lung Cancer [52]	USA	Quantitative, survey	N = 172, current and former smokers in a community	Descriptive statistics: frequency distributions. Bivariate analyses, Pearson correlation analysis, hierarchical multiple linear regression
17. See et al. 2020, ERJ Open Research [53]	Australia	Quantitative, survey	N = 283, ever smokers attending outpatient clinics at three Australian hospitals	Descriptive statistics, chi-squared, t-tests
18. Smits et al. 2018, Health Expectations [54]	UK	Quantitative, survey	N = 1007, general population of adults aged 16 or over in Wales	Multivariate regression
19. Stephens et al. 2019, Lung [55]	USA	Quantitative, web-based national survey	N = 756, general US population	Descriptive statistics, bivariate association, multivariable association
20. Tonge et al. 2019, Health Expectations [56]	UK	Qualitative, semi-structured focus groups	N = 33, screening eligible individuals in Manchester, England	Inductive thematic analysis
21. Simmons et al. 2017, Lung Cancer [57]	USA	Qualitative, focus groups	N = 61, high risk people in one part of Florida and PCPs involved in offering screening	Constant comparative method

### Evidence synthesis

The data from the included studies have been synthesised into a series of themes and sub-themes to summarise and explain the findings. These have been organised into four superordinate themes: Awareness of lung screening, Enthusiasm for lung screening, Barriers to screening, and Facilitators and ways of promoting screening participation. Table 2 indicates which sub-themes are present in each of the 21 studies.

**Table 2:** Themes evident in included studies

	Author, year, journal	1. Ali et al. 2015, BMJ Open (37)	2. Carter-Harris et al. 2017a, Family Practice [38]	3. Carter-Harris et al. 2017b, Health Expectations [39]	4. Delmerico et al. 2014, Lung Cancer (40)	5. Draucker et al. 2019, Health Expectations [41]	6. Greene et al. 2019, Journal of Cancer Education [42]	7. Jonnalagada et al. 2012, Lung Cancer [43]	8. Lowenstein 2019, Lung Cancer [44]	9. Percac-Lima et al. 2019, Journal of Immigrant and Minority Health [45]	10. Quaife et al. 2017, Health Expectations [46]
Themes and sub-themes											
1. Low awareness of lung screening			✓	✓			✓	✓	✓	✓	✓
1.1. Awareness of early detection message				✓		✓	✓		✓		✓
2. Enthusiasm for lung screening				✓		✓	✓		✓	✓	✓
3. Barriers to lung screening											
3.1. Concerns about the test itself				✓				✓	✓	✓	✓
3.2. Fear of a cancer diagnosis/ cancer worry		✓	✓	✓	✓	✓		✓	✓	✓	✓
3.3. Fatalism			✓				✓	✓	✓		✓
3.4. Fear of invasive procedures		✓	✓						✓	✓	✓
3.5. Mistrust of health professionals or services		✓		✓			✓	✓			✓
3.6. Perceived risk of lung cancer (also potential facilitator)		✓			✓	✓		✓		✓	✓
3.7. Smoking-related stigma				✓			✓				✓
3.8. Practical barriers		✓	✓	✓	✓	✓		✓	✓	✓	✓
4.0. Facilitators or suggestions to improve uptake											
4.1. Being health conscious						✓	✓				
4.2. Provider recommendation						✓	✓	✓	✓	✓	✓
4.3. Motivation to quit smoking				✓			✓				✓
4.4. Seeking reassurance				✓			✓				✓

	Author, year, journal	11. Quaife et al. 2018, BMC Cancer [47]	12. Raju et al. 2020, Clinical Lung Cancer [48]	13. Raz et al. 2019, Clinical Lung Cancer [49]	14. Roth et al. 2018, PLoS One [50]	15. Schiffelbein et al. 2020, Journal of Primary Care and Community Health [51]	16. Schnoll et al. 2003, Lung Cancer [52]	17. See et al. 2020, ERJ Open Research [53]	18. Smits et al. 2018, Health Expectations [54]	19. Stephens et al. 2019, Lung [55]	20. Tonge et al. 2019, Health Expectations [56]	21. Simmons et al. 2017, Lung Cancer [57]
Themes and sub-themes												
1. Low awareness of lung screening		✓	✓	✓	✓	✓	✓			✓	✓	✓
1.1. Awareness of early detection message		✓			✓		✓	✓	✓	✓	✓	✓
2. Enthusiasm for lung screening		✓	✓		✓	✓	✓	✓	✓	✓	✓	✓
3. Barriers to lung screening												
3.1. Concerns about the test itself			✓	✓	✓	✓						✓
3.2. Fear of a cancer diagnosis/ cancer worry		✓	✓	✓				✓	✓			✓
3.3. Fatalism		✓							✓	✓		
3.4. Fear of invasive procedures												✓
3.5. Mistrust of health professionals or services		✓			✓							
3.6. Perceived risk of lung cancer (also potential facilitator)		✓	✓	✓		✓	✓		✓	✓		
3.7. Smoking-related stigma		✓		✓								
3.8. Practical barriers			✓	✓		✓		✓		✓		✓
4.0. Facilitators or suggestions to improve uptake												
4.1. Being health conscious					✓		✓				✓	
4.2. Provider recommendation		✓			✓	✓						✓
4.3. Motivation to quit smoking												✓
4.4. Seeking reassurance		✓										

#### Awareness of lung screening

The literature consistently demonstrated low awareness of lung screening (e.g. [51, 57]). Participants were aware of other screening programmes but had often not heard of or been previously screened for lung cancer. However, some studies did explore views among people who had been offered lung screening and so lack of awareness was most applicable in studies of wider, screening eligible populations. For example, in a study by Raz et al., 18.9% of their sample had undergone lung screening, while the remainder had not heard of it nor been offered lung screening [49]. US studies in our review suggested that participants did not discuss lung screening with their providers beyond basic conversations about eligibility and intention to refer, and respondents typically suggested that they were not provided with sufficient information about screening before taking part [44]. A lack of provider awareness of lung screening could also impact on the number of opportunities to be offered screening, as has been shown in a US context [57].

There was a wider awareness of other cancer screening services, and many studies (11 out of 21) reported that participants were aware of the importance of early detection and its impact on survival e.g. [39, 41, 44].

#### Enthusiasm for lung screening

Despite low awareness of lung screening opportunities in the reviewed literature, there was strong support in principle for lung screening, and a broader awareness of the link between early detection and improved survival rates [56]. For example, Lowenstein et al. found in a qualitative study with 42 screening eligible patients that participants were in favour of screening, found it acceptable, and related screening to prevention and early detection [44]. A number of studies also suggested that people sought reassurance or peace of mind from undergoing screening (as discussed in relation to facilitators), although this was complex: some participants reported that this gave them the ‘go ahead’ to continue smoking, while for others it was a motivation to quit [39, 46].

#### Barriers to lung screening

Barriers to screening participation constituted the largest category in our analysis and a number of sub-themes were identified across the included studies.

##### Concerns about the test itself

Concerns about aspects of the screening test itself were reported as barriers to participation in ten studies in this review (see Table 2). The main concerns about undergoing screening were related to unnecessary radiation exposure, over-diagnosis and the risk of a false positive result e.g. [43–45, 57]. For Lowenstein et al's participants, the concerns centred around the psychological harms of false positives, incidental findings and radiation exposure [44]. However, it is important to note that these barriers were considered to be minimal in relation to the potential benefits of early detection. This was echoed in other included studies: Roth et al. reported less than half of participants raised these concerns and believed the benefits of screening to outweigh the harms in their interviews with a screening eligible population, while Quaife et al. reported only one participant being concerned about radiation exposure in a survey and interview study with 184 participants [46, 50].

##### Fear of a cancer diagnosis/ cancer worry

Fifteen included studies reported that fear of cancer diagnosis, or cancer worry, mediated lung screening intentions, as shown in Table 2. Related to these is an avoidance of lung cancer information [37]. A number of these studies reported that their participants did not want to know if they had cancer as an avoidance mechanism for the associated worry of waiting for a screening result and the distress of a cancer diagnosis [40, 41, 49]. There was a perception among some study participants that a lung cancer diagnosis was a ‘death sentence’ [44, 46], although not all studies found a clear association between lung cancer worry and intention to be screened [47]. Notably, a survey by See et al. also found that worry about lung cancer was positively associated with lung screening participation, and so it can be a motivator to taking part for some invitees [53].

##### Fatalism

Evidence of fatalism among screening eligible participants was related to screening participation in a number of studies e.g. [46, 54, 55]. In a mixed methods study of an at-risk population with low socioeconomic status, Quaife et al. found that 20% of their participants felt that they had smoked too long to benefit from lung screening. Further qualitative inquiry elucidated that screening could not get the participants ‘a new pair of lungs’ and they expressed a lack of control over lung cancer, even if they stopped smoking [46].

##### Fear of invasive procedures

There was evidence in included studies for fear of invasive procedures that may be necessary for the screening test itself [38, 44, 57]. Participants in these studies also reported a fear of follow-up investigations, procedures or treatments associated with a lung cancer diagnosis.

##### Mistrust of health professionals and services

A number of included studies (7 out of 21) reported a mistrust in health professionals and services, sometimes related to poor experiences in the past or negative associations with loss of loved ones, leading to avoidance of any interactions or engagement with services e.g. [37, 39, 42, 50]. Quaife et al. described the association of hospitals with a ‘slippery slope towards death’, with a desire to avoid them [46]. Conversely, trust in a referring clinician or physician endorsement were positively associated with screening participation [50], although this could be mediated by smoking status [47].

##### Perceived risk of lung cancer

Perceived risk was reported as a barrier to participation in lung screening (13 of 21 papers) eg [43, 48, 49, 51]. Absence of symptoms of lung cancer and a feeling of being fit and well led to a perception among some study participants that screening is unnecessary e.g. [49, 51].

##### Smoking-related stigma

Perceived stigma related to current or past smoking behaviour was reported as a barrier to participation. For example, complex feelings of shame, self-blame and stigma were found by Greene et al. in their qualitative study of influences on lung screening decision-making among recent participants [42]. Their participants reported being treated unfairly by health care professionals due to their smoking and a failure to understand the cultural connotations of smoking in previous generations.

##### Practical barriers

A number of practical barriers were reported in the included studies. These ranged from issues such as time off from work [57], competing priorities in terms of chronic conditions or caring responsibilities [46], and travel and transport [51]. Cost also posed a barrier to screening participation, as some believed they would have to pay for the test or there would be financial implications of travel or taking time off work [49]. In US studies, concerns and misunderstandings about insurance cover were also reported [48, 55].

#### Facilitators or ways of promoting uptake of lung screening

##### Being health conscious

A concept that appeared to be common among those who had participated in screening and experienced fewer barriers to doing so was a desire to keep ‘on top of’ their health and a stronger sense of self-efficacy [41, 43, 52]. Roth et al. report that having seen friends or family members go through cancer motivated people to monitor their own health and act quickly, again showing that previous negative experiences can lead to either promoting screening behaviour, or screening avoidance [50]. Jonnalanga et al. found a significant association between intention to screen and self-efficacy and have applied the self-regulation model to their findings to interpret behavioural cues for screening [43, 58].

##### Provider endorsement

Intention to screen was influenced by provider recommendation or endorsement in a number of included studies [41, 51, 57]. In the USA, the opportunity to be screened is often reliant on physician referral, and many participants in the US studies indicated that they would go for screening if their primary care physician recommended it [43, 45]. Greene et al. suggest that provider recommendation is especially important if patients have a positive and trusting relationship with health care providers [42], while Quaife et al. highlight patient preferences for GP endorsement and health professional support to answer questions and address concerns about screening in a UK context [46, 47].

##### Motivation for behavioural change and smoking cessation

Participating in lung screening was associated with providing motivation to quit smoking among participants in a small number of the studies in the review [39, 42, 46, 57]. Participants described being given a ‘clean slate’ by a negative lung screen as providing the motivation to quit smoking [42, 46].

##### Seeking reassurance

Further motivation to attend lung screening came in the form of people wanting to ‘see the state of their lungs’ in order to assess whether action was needed (lifestyle change or quitting smoking). Receiving a clear scan was regarded as giving peace of mind that they were not going to develop lung cancer and reassured study participants that they did not have to worry [42].

## Discussion

### Summary

This is the first review to provide a concise summary of the key patient barriers and facilitators to lung screening, comparing the literature and drawing together the evidence. This review includes 21 papers reporting patient perceptions of barriers and facilitators to targeted lung cancer screening using low-dose CT. Although it identified a heterogeneous group of papers with different populations, designs and methodologies, there was strong consistency in the findings, allowing for the aggregation of findings and synthesis into a number of reported themes. While awareness of lung cancer screening is low, support for lung screening and awareness of the benefits of early detection was evident across the included studies. However, the manner in which intention to screen translates to participation in screening is mediated by a number of barriers to action. Psychological barriers to lung screening were commonly reported in the literature and included fear of a cancer diagnosis, fear of invasive procedures, mistrust of health professionals and services, fatalism, perceived stigma, and low perceived risk of lung cancer. Facilitators to lung screening identified in the literature were high perception of risk of lung cancer, being ‘health conscious’, provider recommendation or endorsement, seeking reassurance and motivation for behaviour change i.e. smoking cessation.

### Comparison with wider literature and theory

Low levels of awareness of lung screening in the literature was noted. The relative infancy of lung screening, the low levels of opportunistic lung screening in the USA, and the lack of a national UK programme for lung screening could account for the low level of awareness compared with other forms of screening. Across the wider literature on screening, support for screening is a very common finding [59–61]. However, being in favour of screening is not the single biggest predictor of screening behaviour, as a number of cognitive, behavioural, environmental and system factors converge to influence performed screening behaviours in the gap between intention and behaviour [62].

A number of participants reported concerns about features of the screening test such as radiation exposure and false positives. Comparisons can be drawn with breast screening where women were concerned about the accuracy of the test [63], whereas test-related concerns about bowel screening related more to competency in carrying out the test and disgust handling faeces [59]. While it is important to address lung screening test-related concerns and provide information and opportunity to discuss these where relevant, practical barriers were far outweighed by perceived benefits in the reviewed literature. However, if we consider participation as a set of scales, these concerns could be enough to tip someone into non-participation in combination with other issues.

Fear of a cancer diagnosis was one of the most dominant themes in the literature, coupled with fear of invasive procedures—suggesting that psychological harms associated with cancer worry and waiting for screening results prove significant barriers to screening participation. This mirrors the literature in relation to other screening programmes and decisions to seek help for cancer symptoms, which can help us predict behaviours in relation to lung screening participation [59, 64]. Fatalism was also proposed as a cognitive response to non-participation in screening. Fatalism is a complex response that is likely bound with fear and avoidance and could be something that is entrenched in social discourses of cancer in deprived populations [65, 66].

Related to fear is a mistrust of health professionals and services and perceived stigma or judgement for smoking, which are powerful psychological emotions that can mediate engagement with health services, lead to avoidance, and that are socially patterned [67]. There is a call to understand this more in the Scottish context, and we have conducted some [68] focus group work to gain insight to this and other barriers to lung screening. Two of the authors have conducted some co-design work identifying similar barriers to lung screening using blood biomarkers [69]. Building trust in health systems is therefore key to improving accessibility of services [70] and breaking down some of the perceived conditions on ‘offers’ of health care (and therefore resistance to these offers), as outlined in Dixon-Woods et al. candidacy framework [34].

Perceived risk of lung cancer can also mediate participation in screening depending on a person’s concern that they may be at risk of lung cancer, with risk influenced by factors such as smoking status and symptom recognition and appraisal. Moreover, even among those groups who perceived their risk of lung cancer to be high, factors such as mistrust or fear of diagnosis may still prevent them from taking part. There is also scope for further work to understand the complexities of smoking status and its impact on lung screening decisions, to develop the work of Quaife et al. [46, 47].

Applying psychological theory is helpful here to understand the influence of psychological barriers—such as perceptions of risk, complex fears, and stigma—on screening behaviour. Leventhal’s self-regulation model purports that self-assessed risk and cues to action are underpinned by representations of cancer and the self, linking with other evident themes in the review around cancer worry and narratives of a ‘cancer death sentence’ [58]. However, these cues, or motivations, are only one component of understanding health behaviour as Michie’s behaviour change wheel (encompassing motivation, capability and opportunity) shows, acknowledging both internal and external influences on health behaviours such as screening to be considered when designing an intervention [71].

There was a consistent theme in included studies about the practical barriers to screening participation such as cost, time and travel. Weighing up the significance of practical barriers relative to psychological and behavioural factors is an important consideration in pilot studies of lung screening. See et al. suggest that practical barriers are minor or not ‘deal breakers’ in terms of preventing people from participating in screening, but this was a study conducted among those who participated [53]. Gaining insight to real world scenarios among non-participants is needed for balance in understanding the influence of practical barriers. Issues of access are also likely to be significant for the rural population of Scotland with areas of high deprivation, and work is underway elsewhere in the UK that can provide insight to addressing these issues (e.g. the use of mobile units) in combination with more in-depth, qualitative work [72].

There was less evidence in the review for facilitators to screening but some themes were identified that could influence taking part or help promote screening. Being ‘health conscious’ was identified as positively influencing screening participation and was related to self-regulation and self-efficacy in one study [43]. Wardle et al. encapsulate this issue in their discussion of being health conscious and its links to opportunity, material hardship, adverse experiences, and the resources to think about future health; this has been built into subsequent models of health and screening behaviour [62, 73]. Together with issues of fear and trust, opportunities to be ‘health conscious’ are embedded in wider social structures that go beyond screening to a more upstream public health approach to addressing social inequalities.

Provider recommendation and endorsement were also identified as facilitators to lung screening, which has also been an important factor in promoting bowel screening [59, 74]. This will rely on promoting provider awareness of and support for lung screening. It is unclear, however, how endorsement will translate into action in the context of a lung screening programme [46], and as the discussion of trust highlights, the importance of this relationship should not be undervalued.

Another facilitator to take part in lung screening, to confirm that cancer is not present and to give the individual peace of mind, is seeking reassurance—this has been identified as a motivation for taking part in other screening programmes [75, 76]. However, there is a need to understand the impact of reassurance on future symptom appraisal, help-seeking and repeat screening behaviour [77, 78].

Offers of lung screening can also prompt positive action in relation to other areas of health promotion and lifestyle change, most notably smoking cessation. There is emerging evidence that lung screening can be a motivator to quit smoking and positively impacts smoking cessation rates [79]. Integrated smoking cessation has also been identified as a key component of a lung screening programme to ensure cost effectiveness and long-term health gains [7, 80].

### Strengths and limitations

Our review provides a novel synthesis and insights to evidence from a behavioural science perspective examining how barriers to screening participation may combine in real world situations to explain the gap between screening intentions and screening behaviour.

The review is limited to patient reported barriers and facilitators published and indexed in seven databases over a recent twenty year period, and does not include papers published since mid-2021 or wider implementation issues impacting on lung screening provision. Lung screening is a very fast paced area of current research and so more up to date evidence becomes ever available and may shift the evidence base. Smoking status could impact on the views expressed, e.g. among a group of people attending a smoking cessation class [49], and this variation in position should be considered when interpreting findings.

Implications Synthesising the evidence on patient barriers and facilitators to lung screening, and interpreting these findings in the context of existing evidence and theory, allows us to inform the development of evidence-based interventions, a cornerstone of implementation science, and sustainable healthcare policy and practice [81].

This synthesis will be combined with findings from a focus group study and stakeholder consultation to inform the development of a pilot targeted lung screening intervention to be feasibility tested in Scotland. Implications for a pilot lung screening study are listed in Box 3.

Box 3: Summary and recommendations for overcoming patient barriers to lung screening

- Patient level barriers to lung screening are complex and multi-faceted and combine to push people into non-participation; strategies to promote uptake need to address this complexity
- Psychological barriers including fear, stigma, fatalism and mistrust of services are powerful and steps to address these barriers should be built into the design of public facing information and promotional materials, offering recognition, reassurance and positive messaging
- Provider recommendations and endorsements can engender trust in the screening process so linking with primary care, eg GP letters or brief interventions, are warranted
- Access issues such as distance, transport, cost and time should be factored into the design of a lung screening service to reduce inequalities in uptake, eg covering travel costs or use of mobile units to reduce travel and bring screening into local communities
- Employer support to provide paid time off work to attend screening
- Adoption of theoretical models of screening to understand patient-level barriers to screening is recommended

## Conclusions

Lung cancer screening has been shown to reduce lung cancer mortality through early detection. However, overall uptake of lung screening (where available) has been relatively low and is socially patterned. This review of the literature has provided insight into patient reported barriers and facilitators to participation in lung cancer screening; this provides the opportunity to combine with empirical work to inform future implementation strategies of lung screening pilots, trials, and to help shape future national lung screening programmes.

## Supplementary Information

**Additional file 1. **Study protocol.

## Data Availability

Extracted information and synthesised data is available on reasonable request to the corresponding author.

## Abbreviations

LDCT: Low dose computer tomography
CT: Computer tomography
SES: Socio-economic status
USPSTF: United States Preventative Services Task Force
US: United States (of America)
UK: United Kingdom
