# Supplementary material for: Understanding patient barriers and facilitators to uptake of lung screening using low dose computed tomography: a mixed methods scoping review of the current literature
Source: Respir Res. 2022 Dec 23;23:374. doi: 10.1186/s12931-022-02255-8 (PMC9789658; doi:10.1186/s12931-022-02255-8)
Supplement: Supplementary file 1 — Additional file 1. Study protocol. [file 12931_2022_2255_MOESM1_ESM.doc]

# Additional file 1

# Study Protocol

# How should Scotland respond to the challenge of lung cancer screening? A mixed methods feasibility study

# Work package 1: Identifying and inviting high risk people to primary care lung cancer screening – a scoping review, document analysis and stakeholder consultation exercise

|  | The University of Edinburgh  ACCORD The Queen’s Medical Research Institute 47 Little France Crescent Edinburgh EH16 4TJ |
| --- | --- |
| Protocol authors | **Professor David Weller, Dr Debbie Cavers** |
| Funder | **CSO** |
| Funding Reference Number | **HIPS/19/52** |
| Chief Investigator | **Professor David Weller** |
| Sponsor number | **AC20167** |
| REC Number |  |
| Project registration | **N/A** |
| Version Number and Date | **Version 1, 19.01.2021** |

*.*

# CONTENTS

1.1 BACKGROUND [7](#__RefHeading___Toc59107252)

1.2 RATIONALE FOR STUDY [8](#__RefHeading___Toc59107253)

2 STUDY OBJECTIVES [8](#__RefHeading___Toc59107254)

2.1 OBJECTIVES [8](#__RefHeading___Toc59107255)

2.2 Research questions [8](#__RefHeading___Toc59107256)

3 STUDY DESIGN and METHODS [9](#__RefHeading___Toc59107257)

4 DATA MANAGEMENT [15](#__RefHeading___Toc59107258)

4.1.1 Personal Data [15](#__RefHeading___Toc59107259)

4.1.2 Data Information Flow [15](#__RefHeading___Toc59107261)

4.1.3 Transfer of Data [15](#__RefHeading___Toc59107263)

4.1.4 Data Controller [15](#__RefHeading___Toc59107264)

4.1.5 Data Breaches [15](#__RefHeading___Toc59107265)

5 DATA ANALYSIS [16](#__RefHeading___Toc59107266)

5.1 Focus group analysis [16](#__RefHeading___Toc59107267)

6 ADVERSE EVENTS [16](#__RefHeading___Toc59107268)

7 OVERSIGHT ARRANGEMENTS [16](#__RefHeading___Toc59107270)

7.1 INSPECTION OF RECORDS [16](#__RefHeading___Toc59107271)

7.2 STUDY MONITORING AND AUDIT [16](#__RefHeading___Toc59107272)

8 GOOD CLINICAL PRACTICE [17](#__RefHeading___Toc59107274)

8.1 ETHICAL CONDUCT [17](#__RefHeading___Toc59107275)

8.2 INVESTIGATOR RESPONSIBILITIES [17](#__RefHeading___Toc59107277)

8.2.1 Informed Consent [17](#__RefHeading___Toc59107278)

8.2.2 Study Site Staff [17](#__RefHeading___Toc59107279)

8.2.3 Data Recording [17](#__RefHeading___Toc59107280)

8.2.4 Investigator Documentation [17](#__RefHeading___Toc59107281)

The Principal Investigator will ensure that the required documentation is available in local Investigator Site files. [18](#__RefHeading___Toc59107282)

8.2.5 GCP Training [18](#__RefHeading___Toc59107283)

8.2.6 Confidentiality [18](#__RefHeading___Toc59107284)

8.2.7 Data Protection [18](#__RefHeading___Toc59107285)

STUDY CONDUCT RESPONSIBILITIES [18](#__RefHeading___Toc59107286)

8.3 PROTOCOL AMENDMENTS [18](#__RefHeading___Toc59107287)

8.4 MANAGEMENT OF PROTOCOL NON COMPLIANCE [18](#__RefHeading___Toc59107288)

8.5 SERIOUS BREACH REQUIREMENTS [19](#__RefHeading___Toc59107289)

8.6 STUDY RECORD RETENTION [19](#__RefHeading___Toc59107290)

8.7 END OF STUDY [19](#__RefHeading___Toc59107291)

8.8 INSURANCE AND INDEMNITY [19](#__RefHeading___Toc59107292)

9 REPORTING, PUBLICATIONS AND NOTIFICATION OF RESULTS [20](#__RefHeading___Toc59107293)

9.1 AUTHORSHIP POLICY [20](#__RefHeading___Toc59107294)

10 REFERENCES [20](#__RefHeading___Toc59107295)

# LIST OF ABBREVIATIONS

| **ACCORD** | Academic and Clinical Central Office for Research & Development - Joint office for The University of Edinburgh and Lothian Health Board |
| --- | --- |
| **PI** | Principal Investigator |
| **WP1** | Work package 1 |
| **WP2** | Work package 2 |
| **WP3** | Work package 3 |
| **LDCT** | Low dose computed tomography scan |
| **LLP** | Liverpool Lung Project |
| **PLCO** | Prostate Lung Cervical Ovarian (screening study) |
| **ECLS** | Early Detection of Cancer of the Lung Scotland |
| **CT** | Computer Tomography |
| **SIMD** | Scottish Index of Multiple Deprivation |
| **GP** | General Practice |
| **RCT** | Randomised controlled trials |
| **NHS** | National Health Service |
| **PRISMA-ScR** | Preferred Reporting Items for Systematic Reviews – Scoping Review |
| **UK** | United Kingdom |
| **US** | United States |
| **EMBASE** | Excerpta Medica Database |
| **CINAHL** | Cumulative Index to Nursing and Allied Health Literature |
| **ASSIA** | Applied Social Sciences Index and Abstracts |
| **PsychINFO** | Psychological Information |

# 1 INTRODUCTION

## BACKGROUND

Lung cancer incidence rates in Scotland are among the highest in the world with around 5300 Scots diagnosed each year and 4000 deaths(1). Survival from lung cancer in Scotland is low compared to many other European countries, and rates of co-morbidity are high (2). Despite the Scottish Government’s Detect Cancer Early Programme, outcomes remain poor with, typically, late stage diagnosis,^1^ which results in costly treatment without significant impact on survival. Screening for lung cancer within North American and European studies, using low-dose CT screening (LDCT) suggests diagnosis at an earlier stage and a survival benefit without the risks of ‘over-diagnosis’ (3, 4), and numerous regional pilot studies and trials within the UK are underway (5, 6). Yet there is no wide-spread agreement about how to respond to this emerging evidence; lung screening only works at a population level if it identifies and recruits all, or most of the ‘right people’ to screening (3, 4) – that is, those who are at highest risk from the disease.

There are important unanswered questions in lung cancer screening; for example, while recruitment through primary care is likely to be favoured in the UK, the information in primary care records to enable effective approaches to potentially eligible study subjects may be insufficient to underpin effective risk-based recruitment to screening (7). Further, we do not have a widely accepted process for identifying and recruiting those who stand to gain the most benefit from screening. This is crucial as, in contrast to existing programmes, evidence of benefit from lung cancer screening has only been demonstrated in high-risk groups. A range of strategies have been used to identify appropriate invitees for lung cancer screening. These include questionnaires, use of existing primary care data and the application of risk models such as the Liverpool Lung Project (LLP) (8) and the PLCO_2012_ models (5). Strategies need to be acceptable and feasible, and avoid generating significant extra burden and costs – in particular, they need to avoid over-diagnosis, whereby unacceptable numbers of participants receive investigations and treatments which lead to no health gain.

Lung screening in Scotland needs to accommodate our particular challenges, relating to healthcare disparities, deprivation and rurality. Further it should build on existing work (such as our ECLS study) (9), and facilitate future population-based trials. Importantly, screening shouldn’t reinforce existing inequalities in health through differential uptake; lung cancer in Scotland is more common in socio-economically deprived groups with European Age-Sex Standardised rates of 181.0 in the most deprived SIMD quintile, compared with 56.4 in the least deprived (10). Further, rurality can impact on cancer outcomes (11), so it is important to explore any rural-urban differences in acceptance of lung checks and screening. At present the Scottish Government and the lung cancer clinical and research community are exploring how best to respond to the challenges of lung cancer early detection and screening; a programme of work designed to meet the unique needs of the Scottish population is required – this will complement pilots and trials in England and Europe (12). A careful analysis of feasibility issues, focusing on engaging the ‘right’ individuals in screening, examining access and rurality, and measuring impact on health disparities, will help guide Scotland’s approach to lung cancer screening.

## RATIONALE FOR STUDY

At present we know that, within the target population for lung cancer screening, there are often attitudes and beliefs that can act as a barrier to participation. These include a sense of nihilism about lung cancer and fear over what screening might find (13). Further barriers identified include deprivation and associated issues such as low health literacy and access to services due to rurality or perceived candidacy and a personal focus on future health(13, 14).

A scoping review of the literature on lung screening uptake, with a focus on strategies to reduce disparities in those who accept invitations, will illuminate the most effective strategies for engaging high risk populations in the lung screening process. We will combine this with relevant information from a stakeholder consultation workshop, review of existing lung cancer screening trial study documentation and focus group interviews with heavy smoking members of the Scottish population living in areas of rurality and/or high deprivation.

# STUDY OBJECTIVES

## OBJECTIVES

We propose a mixed methods study to ascertain a) how well we can identify at risk individuals in primary care settings, and b) acceptability and feasibility in the Scottish population. This work will complement and inform any concurrent and future population-based trials of lung CT screening.

This protocol reports on Work Package 1 of a larger feasibility study that will test a lung cancer screening intervention in the Scottish population. The components in this work package include consultation and service evaluation comprising of 1) a scoping review; 2) a stakeholder consultation; 3) a study document review, and research involving 4) a focus group study with high risk individuals.

## Research questions

1. Which method(s) of identifying people at the highest risk of lung cancer (including the application of risk models) is likely to be the most effective in primary care?
2. What sources of information (GP records, smoking cessation clinics, patient questionnaires etc.) are need to gain a comprehensive picture of lung cancer risk?
3. Which methods are most effective at equitably recruiting high risk people to lung screening?

*Component 4, focus group study, is the subject of this ethical review.*

# STUDY DESIGN and METHODS

1. Scoping review

An initial scoping review will be employed to examine how best to identify those most at risk of lung cancer in primary care, how best to feasibly recruit them to lung cancer screening and how to target inequality at the heart of lung cancer and lung cancer screening. Two separate mixed methods rapid scoping reviews will focus on 1) existing tools/approaches/models to assess lung cancer risk and experience to date in implementing these in primary care settings and 2) Rates of and barriers to invitations to participate in lung screening. We will adopt a scoping review methodology, which aims to provide high quality evidence in a short timeframe (15-17). Scoping methodology was considered appropriate to give insight to a broader topic area to understand the landscape and map key concepts, rather than focusing on a very focused research question looking at effectiveness (15). We will follow Arksey and O’Malley’s five-step process including identifying the research question, identifying the studies, study selection, charting the data, and collating, summarising and reporting the results. We will also reflect on the limitations of the approach and consider suggested enhancements (16, 17).

*Identifying and selecting studies*

We will engage with specialist librarian input to develop the review methodology, including the full search strategy of terms, target databases and other forms of identifying articles. Proposed databases include Medline, EMBASE, CINAHL, PsycINFO, ASSIA, Web of Science, Sociological Abstracts, and SCOPUS. A preliminary list of search terms are listed in Box 1 for each of the reviews. Articles in each review will be subject to inclusion and exclusion criteria that will be finalised after the searches have taken place and the relevant criteria become apparent with greater familiarity with the literature, in line with the suggested methodology (15). Basic inclusion and exclusion criteria are detailed in Box 1. Articles will be subject to a three stage screening process: 1) initial title screening to remove clearly irrelevant studies, 2) title and abstract screening to select relevant studies, and 3) full text screening to select the final studies for inclusion in the review. In addition, a snowball approach following up on key references and searching the reference sections of included articles will be implemented to find additional relevant studies not identified by the database searches.

*Dimensions of interest*

The first review will focus on mechanisms of recruitment to lung cancer screening. Strategies typically require 2 components: 1) a means of stratifying patients according to their lung cancer risk, then 2) implementing these risk models in a recruitment process. We are particularly interested in recruitment strategies which involve primary care. So this first review will look at existing tools/approaches/models to assess lung cancer risk and experience to date in implementing these - especially in primary care settings. The review will also look at mechanisms of recruitment – e.g. postal, opportunistically (e.g. vans in supermarket car parks), approaches from primary care etc.

The second review will focus on uptake rates of invitations to participate in lung screening. To date there have been a small number of RCTs and some pilot projects which have tested a variety of recruitment strategies. Ideally uptake rates will be examined by method of recruitment, age, gender, ethnicity, rurality and deprivation.

| Box 1: Search terms |  |
| --- | --- |
| Review 1 | Review 2 |
| Lung cancer OR lung neoplasm  AND  Screening  AND  risk  OR  High risk  assess$  model$  tools  Primary care  General practice  Implement$  Algorithim  Strateg$  Over diagnosis  Valid$ | Lung cancer OR lung neoplasm  AND  Screening  AND  Barriers  OR  Facilitat$  Health literacy  Socioeconomic  Deprivation  Candidacy  Access$  Participation  Eligibil$  Disprarit$ |

Quality assessment is not a core part of the scoping review methodology and will not be undertaken as part of this review.

*Extracting and charting data*

For both reviews, relevant data will be extracted and added to an excel pro-forma with separate fields to collect relevant information related to review questions. This will include details of the study authors, design and setting as well as findings relating to characteristics of existing tools used to identify at risk patients to lung cancer screening for review 1 and barriers, facilitators and patient and professional experiences of identifying patients for lung cancer screening. The fields in the pro-forma will be developed after final selection of included studies when the relevant information becomes available.

Inclusion criteria:

- Articles focusing on areas denoted in the dimensions of interest, iteratively refined
- Study designs that are qualitative, quantitative, mixed methods, systematic reviews, clinical trials.
- Articles published in English

Exclusion criteria:

- Articles not meeting the above inclusion criterion.
- Articles published from 2015 (review 1) and 2000 (review 2)
- Articles focusing on effectiveness of lung screening trials

*Collating, summarising and reporting the results*

Depending on the form and content of the data identified from the review, it will be subject to appropriate and proportionate summarising. Possible approaches include narrative or thematic synthesis or qualitative content analysis (18, 19). Reporting of the scoping review will follow the PRISMA checklist extension for scoping reviews PRISMA-ScR (20).

*Dissemination*

Findings from the scoping review will form part of report to the Scottish Government Chief Scientist’s Office. In addition, they will be presented at internal, national and international seminars and conferences as well as published in relevant, peer-reviewed scientific journals.

1. Stakeholder consultation

We will host a one-off stakeholder consultation workshop to bring together a range of people invested in lung cancer screening. Lung cancer screening is typically driven by a very broad range of stakeholders, and it is important at the outset of the broader feasibility study to seek their input on the design and implementation of the subsequent work packages of the project. This is a consultation exercise constituting service evaluation, rather than a research study.

*Identifying and contacting stakeholders*

A range of stakeholders will be identified using the expertise of the multi-disciplinary project team, including clinicians and academics working in lung cancer care and screening development. The workshop will include representation from patient groups, Scottish Government, health policy and planning, NHS Scotland National Specialist Health Services and Screening team, and health provider groups, including imaging, primary care, pathology, thoracic surgery and radiotherapy. We will use existing contacts to invite people via a formal electronic letter of invitation to take part in the consultation workshop.

*Patient involvement*

Patient representation and contribution is key to this event to ensure discussions are meaningful and relevant to patient concerns. The research team will use existing contacts with patient groups through related lung screening research, to invite members to join the workshop. As above, patients will be sent a formal electronic letter of invitation to attend the workshop and arrangements will be made via email. Patients are being invited to join the workshop as partners in this consultation exercise rather than participants in a research study. We will not be collecting research data that is subject to formal analysis. Patient representatives will be invited to join the project steering group to continue to contribute to work packages of the study.

*The workshop*

A one-off workshop will take place, planned for spring 2021, to bring together the identified stakeholders. The preferred option is to host the workshop face to face at an accessible university venue. However, due to unknown restrictions related to the coronavirus pandemic, a virtual event using video conferencing software Microsoft Teams may take its place depending on restrictions on gatherings and movement at the time, or a specific venue to allow extra space for social distancing will be chosen if appropriate. The workshop will address:

- Strategies to identify people as high risk of developing lung cancer

- Features of successful and unsuccessful recruitment strategies for high risk people into screening programmes

- Consideration of practical and implementation issues (confounded by the coronavirus pandemic)

- Keeping the patient experience and patient-centred care at the heart of strategies developed.

A trained facilitator will lead the workshop and members of the research team will attend to assist with facilitating small groups. The format and programme for the workshop has been developed through discussion among the research team, drawing on their expertise of the topic area and modelling the structure and approach on similar events run as part of relevant studies in the past. It will involve up to 24 people in up to 4 small groups, each led by a project team facilitator. If the event is held online, this will involve breakout rooms on Microsoft Teams. After a whole group introduction and overview of the project and the purpose of the workshop, stakeholders will break into small groups (divided into patients, Scottish government, third sector and NHS staff) for in-depth discussion before reconvening to feedback the outputs of their groups and whole group discussion. Key points will be summarised by the overall facilitator, including consensus points and

issues for debate. The workshop will be recorded with the consent of those attending to allow the research team to capture all that is said and listen back to the recording to summarise the key issues.

*Translation*

The information gathered at the workshop will not be subject to formal analysis. The recorded discussion will be listened to in combination with notes taken and key relevant features identified. These will inform the subsequent design and implementation of a feasibility study to test the identification and recruitment of high risk individuals to a lung health check and screening programme, as part of work packages 2 and 3, for which funding has been secured. Work packages 2 and 3 will be subject to future ethics review and approval before the intervention commences.

1. **Document review**

Review of study documents included in existing lung cancer screening trials, past and present, will be undertaken to ensure existing successful strategies for recruiting hard to reach high risk groups to lung screening are incorporated into our intervention design and implementation. This component is classed as service evaluation and not research. This method is part of the triangulation of multiple data sources in order to optimise the intervention (21).

*Study materials*

We will include invitation materials from our own ECLS study, the Yorkshire randomised control trial, various pilots around the UK and studies in Europe and North America. These will be identified via the expert knowledge of the research team working in lung cancer screening in addition to the scoping review. We will be looking for features and strategies which particularly target low health literacy and low socioeconomic groups - that is those in whom we expect low acceptance rates, but biggest potential benefit.

*Document retrieval*

As well as including materials from studies that have been undertaken by members of the research team, we will make contact via email with clinical teams around the UK, Europe and the US to ask permission to have access to their study materials. With their agreement, we will ask for study teams to send their materials electronically along with any feedback on barriers and facilitators to implementing lung screening trials or interventions. All study teams will be fully acknowledged for their contribution to this consultation exercise in any published materials.

*Document analysis and synthesis*

Study materials will be collated according to document type. Relevant material will be examined and compared across documents. Established methods of document analysis will be employed to appraise and synthesise extracted information (21). Relevant passages of text will be extracted and recorded in a pro-forma set up on Microsoft Excel and based on the key features of the document retrieved. Using a thematic analysis approach used in qualitative research (22, 23), categories will be developed based on key themes identified in the data in addition to pre-defined codes relating to the features of the documents we are interested in, to include: trial design, recruitment strategies, features of participant information materials, challenges and solutions to recruitment difficulties. We will evaluate the evidence in the context of the ‘success’ of the trials in terms of effective feasibility parameters and use these to inform the development of study materials for work packages 2 and 3 of the project.

1. **Focus Group Study**

We will undertake a series of focus group interviews with heavy smokers living in rural, urban and deprived areas of Scotland to ascertain their views on barriers to lung cancer screening. Focus groups are considered an appropriate method for stakeholder engagement (those eligible for and thus directly affected by the screening programme) to understand how they perceive a specific experience such a lung cancer screening and allow the sharing and development of ideas (24). Findings from the focus groups will feed into the development of recruitment strategies and feasibility study materials in the next work package of the project.

*Identifying participants*

We will work closely with research company Taylor MacKenzie (TM), a Scottish-based company that specialise in qualitative research, to identify members of the public eligible to take part in the focus groups. We have experience of working successfully with TM on previous projects exploring public views and perceptions (25). We will provide a screening questionnaire to allow Taylor MacKenzie to identify eligible participants from their extensive database of people willing to take part in health, social or marketing research.

*Recruitment and sampling*

We aim to recruit up to 24 people to take part in four separate focus groups of up to 6 people, considered to be an appropriate number to identify a range of views (26). Interested people respond to recruitment notices posted on TM’s mailing list and social media pages and subsequent participants can be recruited using a snowball approach for hard to reach groups by asking them to share the advert with friends whom they think would be interested in taking part. We will seek to recruit participants based on the following inclusion and exclusion criteria:

Inclusion criteria

- Men and women
- Age 45-70 (inclusive)
- Current residents in Scotland
- Self-identify as smoker or recent quitter (within the last 2 years)
- Able to undertake focus group interview in English
- Willing to discuss their views on lung health checks and screening

Exclusion criteria

- Lacking capacity to give informed consent
- Never smoker
- Smoker who quit more than 2 years ago
- Non-English speakers, preventing them from comfortably taking part in a discussion

Those who respond will be provided with a study information sheet by TM and given 7-14 days to consider taking part. TM will screen those wish to take part for eligibility for inclusion in the study using criterion provided by the research team using information on their database (e.g. postcode for SMID, occupation) and by speaking to potential participants directly (smoking status). A list of eligible people will be drawn up and, together with their eligibility information, added to a password protected file on a secure portal. The researcher will be given access to this portal to download the password protected file to a secure university network drive. The researcher will then contact those interested to discuss the study before they agree to a focus group interview at a pre-set date and time. Contact details of those who do not wish to take part at this stage will be deleted. Participants will be offered a financial reimbursement of £60 for their time, paid via TM.

*Focus groups and consent*

A total of three focus groups will be conducted with 6-8 people in each group. Focus groups will be arranged face to face at a suitable venue, or virtually using the online video conferencing platform Microsoft Teams, if coronavirus restrictions still apply at the time of the interviews.

Microsoft Teams requires an internet connection and participants will follow a web link to access the private room. Participants will be asked to sign a consent form or verbal consent will be agreed with each participant at the beginning of the focus group, depending on whether or not the group is face to face or online. Verbal consent will be recorded and held as a separate file on a password protected encrypted university laptop in line with University of Edinburgh policy. Focus groups will be facilitated by the researcher, following specific focus group guidance and training. They are anticipated to last approximately 1-1.5 hours, with one group specifically with those living in a rural area and the other two urban groups. The format and content of the focus groups has been developed by a sub-group of the research team with expertise in the behavioural aspects of cancer screening. They will explore views on lung health checks and cancer screening, with a particular focus on perceived barriers to taking part (including psychological and emotional barriers: fear, stigma, trust of medical professionals and health services), personal resources to facilitate screening (access to transport, time, health efficacy, orientation to the future and health literacy), understanding of the process and input to what a good screening programme would look like (information and awareness, location, acceptability of procedures, preference for who will deliver the lung health check). With consent, focus groups will be recorded for later transcribing and analysis (detailed in section 5).

**Ethical approvals**

As work package 1 does not involve NHS patients, and only the focus group study involves research participants, NHS ethical approval is not required. Internal ethical approval from the Edinburgh Medical School Ethics Committee (EMREC) will be secured before commencing components two, three and four of this work package.

**Work package 1 Timeline**

| **TASKS** |  | | | | | |
| --- | --- | --- | --- | --- | --- | --- |
|  | **Month 1** | **2** | **3** | **4** | **5** | **6** |
| **1.1 Sponsorship, Ethics, R&D approvals** |  |  |  |  |  |  |
| **1.2 Rapid scoping reviews** |  |  |  |  |  |  |
| Develop search strategy and execute searches |  |  |  |  |  |  |
| Screen studies for inclusion |  |  |  |  |  |  |
| Data extraction, analysis and reporting |  |  |  |  |  |  |
| **1.3 Stakeholder workshop** |  |  |  |  |  |  |
| Identify and invite stakeholders, arrange workshop |  |  |  |  |  |  |
| Summarise key elements, reporting |  |  |  |  |  |  |
| **1.3 Document analysis** |  |  |  |  |  |  |
| Identify and contact trialists, retrieve documents |  |  |  |  |  |  |
| Document analysis and reporting |  |  |  |  |  |  |
| **1.4 Focus group study of heavy smokers** |  |  |  |  |  |  |
| Recruitment with TM and conduct focus groups |  |  |  |  |  |  |
| Analysis and reporting |  |  |  |  |  |  |
| **1.5 Design and development of materials for WP2** |  |  |  |  |  |  |

# DATA MANAGEMENT

### Personal Data

### The following personal data will be collected as part of the stakeholder consultation and document analysis:

- Names and email addresses

The following information will be collected as part of the focus group research:

- Names, postal and email address
- Age
- Smoking status and history (due to a link between heavy smoking and lower lung screening participation and poorer outcomes)
- Education level (due to an interest in health literacy and deprivation)

### Data Information Flow

### Personal information will be kept for the duration of the study (24 months). Age, smoking status and education level will not be linked with names and addresses. Names and addresses will be kept for up to a year afterward the end of the study to allow for contact to be made with people to provide a summary of the findings.

### Transfer of Data

Data collected or generated by the study (including personal data) will not be transferred to any external individuals or organisations outside of the Sponsoring organisation(s).

For the focus group study, participant details will be shared with the study team by Taylor McKenzie, the research agency. This will be done using a password protected file held in a secure portal and access details will be given to the research team.

### Data Controller

The University of Edinburgh and NHS Lothian are joint data controllers along with any other entities involved in delivering the study that may be a data controller in accordance with applicable laws (e.g. the site).

### Data Breaches

Any data breaches will be reported to the University of Edinburgh and NHS Lothian Data Protection Officers who will onward report to the relevant authority according to the appropriate timelines if required.

# DATA ANALYSIS

Information gathered from the stakeholder consultation and document analysis are not classed as research and will not be subject to formal data analysis

## Focus group analysis

Focus groups will be transcribed verbatim by 1st Class Secretarial, a private company that have a confidentiality agreement with the University of Edinburgh, and subject to thematic analysis to derive applied recommendations for work packages 2 and 3 (22). Thematic analysis is the most commonly used approach considered appropriate here to derive key themes and ideas from the group discussions, taking the context into account (24). Thematic analysis will account for the group interview dynamic while enabling the researcher to follow guidance specific to focus group interviewing and its impact on analysis (27, 28).

# ADVERSE EVENTS

# The risk of an adverse event during the stakeholder workshops and focus groups is considered to be minimal. There is no anticipated risk to the personal safety of the researchers, collaborators or participants, particularly if online meetings are held. There is a small risk of emotional distress for participants in the focus groups as they discuss diagnosis of lung cancer generally. The researchers will use a participant-focused approach to questions and respond to verbal and non-verbal cues. In the event that someone does become upset, the focus group will be paused and individuals given the options to continue or terminate their involvement.

# OVERSIGHT ARRANGEMENTS

## INSPECTION OF RECORDS

Investigators and institutions involved in the study will permit study related monitoring and audits on behalf of the sponsor, REC review, and regulatory inspection(s). In the event of audit or monitoring, the Investigator agrees to allow the representatives of the sponsor direct access to all study records and source documentation.

## STUDY MONITORING AND AUDIT

## The ACCORD Sponsor Representative will assess the study to determine if an independent risk assessment is required.  If required, the independent risk assessment will be carried out by the ACCORD Quality Assurance Group to determine if an audit should be performed before/during/after the study and, if so, at what frequency.

Risk assessment, if required, will determine if audit by the ACCORD QA group is required. Should audit be required, details will be captured in an audit plan. Audit of Investigator sites, study management activities and study collaborative units, facilities and 3^rd^ parties may be performed.

# GOOD CLINICAL PRACTICE

## ETHICAL CONDUCT

The study will be conducted in accordance with the principles of the International Conference on Harmonisation Tripartite Guideline for Good Clinical Practice (ICH GCP).

## Before the study can commence, all required approvals will be obtained and any conditions of approvals will be met.

## INVESTIGATOR RESPONSIBILITIES

The Investigator is responsible for the overall conduct of the study at the site and compliance with the protocol and any protocol amendments. In accordance with the principles of ICH GCP, the following areas listed in this section are also the responsibility of the Investigator. Responsibilities may be delegated to an appropriate member of study site staff.

### Informed Consent

The Investigator is responsible for ensuring informed consent is obtained before any protocol specific procedures are carried out. The decision of a participant to participate in research is voluntary and should be based on a clear understanding of what is involved.

Participants must receive adequate oral and written information – appropriate Participant Information and Informed Consent Forms will be provided. The oral explanation to the participant will be performed by the Investigator or qualified delegated person, and must cover all the elements specified in the Participant Information Sheet and Consent Form.

The participant must be given every opportunity to clarify any points they do not understand and, if necessary, ask for more information. The participant must be given sufficient time to consider the information provided. It should be emphasised that the participant may withdraw their consent to participate at any time without loss of benefits to which they otherwise would be entitled.

The Investigator or delegated member of the research team and the participant will sign and date the Informed Consent Form(s) to confirm that consent has been obtained. The participant will receive a copy of this document and a copy filed in the Investigator Site File (ISF) and participant’s medical notes (if applicable).

### Study Site Staff

The Investigator must be familiar with the protocol and the study requirements. It is the Investigator’s responsibility to ensure that all staff assisting with the study are adequately informed about the protocol and their study related duties.

### Data Recording

The Principal Investigator is responsible for the quality of the data recorded.

### Investigator Documentation

### The Principal Investigator will ensure that the required documentation is available in local Investigator Site files.

### GCP Training

For non-CTIMP (i.e. non-drug) studies all researchers are encouraged to undertake GCP training in order to understand the principles of GCP. However, this is not a mandatory requirement unless deemed so by the sponsor.  GCP training status for all investigators should be indicated in their respective CVs.

### Confidentiality

All study records must be identified in a manner designed to maintain participant confidentiality. All records must be kept in a secure storage area with limited access. The Investigator and study site staff involved with this study may not disclose or use for any purpose other than performance of the study, any data, record, or other unpublished information, which is confidential or identifiable, and has been disclosed to those individuals for the purpose of the study. Prior written agreement from the sponsor or its designee must be obtained for the disclosure of any said confidential information to other parties.

### Data Protection

All Investigators and study staff involved with this study must comply with the requirements of the appropriate data protection legislation (including the General Data Protection Regulation and Data Protection Act) with regard to the collection, storage, processing and disclosure of personal information.

Computers used to collate the data will have limited access measures via user names and passwords.

Published results will not contain any personal data and be of a form where individuals are not identified and re-identification is not likely to take place

# STUDY CONDUCT RESPONSIBILITIES

## PROTOCOL AMENDMENTS

Any changes in research activity, except those necessary to remove an apparent, immediate hazard to the participant in the case of an urgent safety measure, must be reviewed and approved by the Chief Investigator.

Amendments will be submitted to a sponsor representative for review and authorisation before being submitted in writing to the appropriate REC for approval prior to participants being recruited into an amended protocol.

## MANAGEMENT OF PROTOCOL NON COMPLIANCE

Prospective protocol deviations, i.e. protocol waivers, will not be approved by the sponsors and therefore will not be implemented, except where necessary to eliminate an immediate hazard to study participants. If this necessitates a subsequent protocol amendment, this should be submitted to the REC for review and approval if appropriate.

Protocol deviations will be recorded in a protocol deviation log and logs will be submitted to the sponsors every 3 months. Each protocol violation will be reported to the sponsor within 3 days of becoming aware of the violation. All protocol deviation logs and violation forms should be emailed to [QA@accord.scot](mailto:QA@accord.scot)

Deviations and violations are non-compliance events discovered after the event has occurred. Deviation logs will be maintained for each site in multi-centre studies. An alternative frequency of deviation log submission to the sponsors may be agreed in writing with the sponsors.

## SERIOUS BREACH REQUIREMENTS

A serious breach is a breach which is likely to effect to a significant degree:

(a) the safety or physical or mental integrity of the participants of the research; or

(b) the scientific value of the research.

If a potential serious breach is identified by the Chief investigator, Principal Investigator or delegates, the co-sponsors (seriousbreach@accord.scot) must be notified within 24 hours. It is the responsibility of the co-sponsors to assess the impact of the breach on the scientific value of the trial, to determine whether the incident constitutes a serious breach and report to research ethics committees as necessary.

## STUDY RECORD RETENTION

All study documentation will be kept for a minimum of 3 years from the protocol defined end of study point. When the minimum retention period has elapsed, study documentation will not be destroyed without permission from the sponsor.

## END OF STUDY

The end of study is defined as the last participant’s recruitment date.

The Investigators or the co-sponsor(s) have the right at any time to terminate the study for administrative reasons.

The end of the study will be reported to the REC and co-sponsors within 90 days, or 15 days if the study is terminated prematurely. The Investigators will inform participants of the premature study closure and ensure that the appropriate follow up is arranged for all participants involved. End of study notification will be reported to the co-sponsors via email to [resgov@accord.scot](mailto:resgov@accord.scot)

A summary report of the study will be provided to the REC within 1 year of the end of the study.

## INSURANCE AND INDEMNITY

The co-sponsors are responsible for ensuring proper provision has been made for insurance or indemnity to cover their liability and the liability of the Chief Investigator and staff.

The following arrangements are in place to fulfil the co-sponsors' responsibilities:

- The Protocol has been designed by the Chief Investigator and researchers employed by the University and collaborators. The University has insurance in place (which includes no-fault compensation) for negligent harm caused by poor protocol design by the Chief Investigator and researchers employed by the University.
- Sites participating in the study will be liable for clinical negligence and other negligent harm to individuals taking part in the study and covered by the duty of care owed to them by the sites concerned. The co-sponsors require individual sites participating in the study to arrange for their own insurance or indemnity in respect of these liabilities.
- Sites which are part of the United Kingdom's National Health Service will have the benefit of NHS Indemnity.
- Sites out with the United Kingdom will be responsible for arranging their own indemnity or insurance for their participation in the study, as well as for compliance with local law applicable to their participation in the study.

# REPORTING, PUBLICATIONS AND NOTIFICATION OF RESULTS

## AUTHORSHIP POLICY

Ownership of the data arising from this study resides with the study team.

# REFERENCES
